# A Two-Stage Algorithm for Origin-Destination Matrices Estimation Considering Dynamic Dispersion Parameter for Route Choice

**DOI:** 10.1371/journal.pone.0146850

**Published:** 2016-01-13

**Authors:** Yong Wang, Xiaolei Ma, Yong Liu, Ke Gong, Kristian C. Henricakson, Maozeng Xu, Yinhai Wang

**Affiliations:** 1 School of Management, Chongqing Jiaotong University, Chongqing, China; 2 Department of Civil and Environmental Engineering, University of Washington, Seattle, Washington, United States of America; 3 School of Transportation Science and Engineering, Beijing Key Laboratory for Cooperative Vehicle Infrastructure, Systems, and Safety Control, Beihang University, Beijing, China; 4 Jiangsu Province Collaborative Innovation Center of Modern Urban Traffic Technologies, SiPaiLou #2, Nanjing, Jiangsu, China; Tianjin University, CHINA

## Abstract

This paper proposes a two-stage algorithm to simultaneously estimate origin-destination (OD) matrix, link choice proportion, and dispersion parameter using partial traffic counts in a congested network. A non-linear optimization model is developed which incorporates a dynamic dispersion parameter, followed by a two-stage algorithm in which Generalized Least Squares (GLS) estimation and a Stochastic User Equilibrium (SUE) assignment model are iteratively applied until the convergence is reached. To evaluate the performance of the algorithm, the proposed approach is implemented in a hypothetical network using input data with high error, and tested under a range of variation coefficients. The root mean squared error (RMSE) of the estimated OD demand and link flows are used to evaluate the model estimation results. The results indicate that the estimated dispersion parameter theta is insensitive to the choice of variation coefficients. The proposed approach is shown to outperform two established OD estimation methods and produce parameter estimates that are close to the ground truth. In addition, the proposed approach is applied to an empirical network in Seattle, WA to validate the robustness and practicality of this methodology. In summary, this study proposes and evaluates an innovative computational approach to accurately estimate OD matrices using link-level traffic flow data, and provides useful insight for optimal parameter selection in modeling travelers’ route choice behavior.

## Introduction

Urban sprawl and population growth have resulted in increasingly severe traffic congestion in major cities around the world. City planners and decision makers have recognized the need for comprehensive traffic management strategies to meet the challenges of rapidly evolving built environments and population demographics. Effective transportation polices and control measures can improve traffic safety and quality of service, as well as promoting economic development and reducing air pollution. Obtaining origin-destination (OD) traffic demand matrix in low-cost and high-accuracy manner not only becomes a problem transportation science, but also draws attentions from many scholars in various scientific fields. For example, researchers in statistical physics and complex systems recently proposed a number of novel methods to estimate OD matrix directly from population data [1, 2, 3, 4, 5, 6]. Reliable OD matrix estimation can provide critical insight for traffic management, operations, and urban planning efforts to mitigate congestion [7, 8]. Thus, a reliable OD matrix estimation method is indispensable for both transportation planners and traffic engineers.

A number of approaches have been developed for estimating OD matrices in the past several decades [9, 10, 11, 12, 13, 14, 15, 16, 17, 18, 19, 20]. Compared with conventional survey-based method, data-driven OD estimation methods relying on link-level traffic flow measurements require less effort and offer significantly reduced time and cost for data acquisition and processing. For such methods, observed traffic flows at key points throughout the network should be known as prior information for OD matrix initialization.

Past research on this topic has considered range of different optimization methods, including entropy maximizing estimators [21, 22], maximum likelihood estimation [23], Bayesian inference estimation [24], generalized least squares (GLS) [9, 10, 25] to estimate OD demands. Entropy maximizing estimators are used to maximize the spread of trip distributions on all available paths (routes) where the observed traffic flows are used as the only information (i.e. without a target trip matrix). Maximum likelihood estimation aims to maximize the likelihood of the closeness between target OD matrix and estimated OD matrix. In the Bayesian inference approach, the target OD matrix is a prior probability function of the estimated OD matrix on a basis of observed traffic count data. The GLS estimator is a robust and efficient linear unbiased estimator, which can solve the estimation of OD matrix by minimizing the Weighted Euclidean Distances (WED) between the target data and the solution data.

User equilibrium (UE) assignment models are commonly used to obtain path choice behavior based on the estimated OD demand. Deterministic UE assignment models assume that all users have access perfect information about the generalized link travel costs, and select a route with the lowest perceived travel cost [26]. Beckman [27] formulated the UE assignment model by assuming that the OD demands are a function of level of service. A combined distribution and assignment model which relies on link-level traffic flow data was presented by Fisk and Boyce [28], and extended by Lam and Huang [29] to address multiclass-user transportation networks. Fisk [30, 31] proposed a combined entropy maximizing model with UE constraints. Yang et al. [11] integrated the GLS technique with a UE traffic assignment model for OD matrix estimation, presented in the form of a convex bi-level optimization problem. Summaries of the more recent contributions to UE-based traffic assignment are provided in Han [32], Lu et al. [33], Inoue and Maruyama [34], Kumar and Peeta [35].

The stochastic user equilibrium (SUE) principle allows the perceived cost to vary between individuals in a heterogeneous population, which can be seen as a more realistic approach than deterministic UE [15, 36], in which the perceived travel costs cannot vary between travelers. The probit SUE was first formulated as a generalization of user equilibrium by Daganzo and Sheffi [37], and developed by Sheffi and Powell [38] as a mathematical programming problem. Liu and Fricker [39] presented a two-stage SUE approach to estimate OD matrices and the probit dispersion parameter in an iterative manner. Yang et al. [15] improved on the methods described in Liu and Fricker by incorporating link traffic flows and travel cost obtained using logit-based SUE traffic assignment. Meng et al. [40] presented a linearly constrained model and solution algorithm for the probit SUE problem with fixed demand and separable link travel time functions. This modeling approach was extended in Meng et al. [41] using elastic demand and non-separable link travel time functions. Time-dependent traffic assignment can be also formulated as a multinomial logit model [42, 43, 44], and this has become one of the most common methods for SUE-based traffic assignment [45, 46, 47]. In a fixed-point formulation, fixed target demands or link flows are used to establish model based on UE and SUE principles [13, 14, 19].

In the multinomial logit model formulation, the link choice probability is a function of a dispersion parameter *θ* [16], which describes road users’ perception of travel costs. Though the dispersion parameter *θ* is predetermined in many previous studies [14, 36, 45, 46, 47, 48], here we assume that this value should be allowed to change with traffic conditions. In addition, Lo and Chan [16] proposed a maximum likelihood procedure for simultaneously estimating the OD matrix and the dispersion parameter *θ*, while the link choice proportions and link flows can be further calculated based on the maximum likelihood estimators of OD matrix and *θ*. Compared with the previous studies, the main contributions of this paper lie in: (1) A fixed-point model is formulated with a dynamic dispersion parameter *θ*, where the estimation of link choice proportions is integrated into the optimization procedure; (2) A GLS estimator is utilized to train this model, and the link choice proportions can be simultaneously calculated based on the OD matrix and dispersion parameter through a multinomial logit model; (3) A two-stage iterative algorithm is presented to refine the OD matrix and dispersion parameter estimates, and Sequential Quadratic Programming (SQP) from the extended quasi-Newton method is applied in the two-stage algorithm process[49].

The remainder of this paper is organized as follows: In Section2, relevant notation, definitions, and model formulations are presented, followed by a link choice proportion approach to calculate the observed link flow using a true OD matrix. A two-stage algorithm is described in Section 3, along with model implementation details. The performance of the proposed approach is tested in a hypothetical network, and a sensitivity analysis is conducted using a range of variation coefficients. Results are presented and compared with those obtained through other established OD estimation methods. In section 4, results are presented for a real-world network using loop detector data in the city of Seattle, WA to demonstrate the practicality of the proposed approach. Finally, conclusions are summarized in Section 5.

## Model Formulation

### Related Notations and Definitions

The notation and parameter definitions used throughout the paper are as follows:

*K* the set of network links *k* ∈ *K*, where *T* denotes the total number of links
*L* the set of observed links *l* ∈ *L*, where Γ denotes the number of observed links
*J* the set of OD pairs *j* ∈ *J*, where *τ* indicates the total number of OD pairs
*A* the set of paths connecting the OD pair *j*, *a* ∈ *A*
*c*_*k*_ travel cost of link *k*
c_*rj*_ travel cost of path *r* connecting the OD pair *j*
*t*_*k*_ the free flow travel time of link *k*
*C*_*k*_ the capacity of link *k*
*α*_*k*_ the performance function parameter of link *k*
*β*_*k*_ the exponential value of link *k*’s performance function
*M* the set of nodes in the network
*f* the vector for estimated link flows, where *f*_*k*_ is the estimated flow for link *k*
$\overset{\wedge }{f}$ the vector for observed link flow, where ${\overset{\wedge }{f}}_{l}$ is the observed link flow for link *l*
*d* the estimated OD vector matrix, where *d*_*j*_ is the *j*th element of *d* for OD pair *j*
$\bar{d}$ the target OD vector matrix, where ${\bar{d}}_{j}$ is the *j*th element of $\bar{d}$ for OD pair *j*
$\tilde{d}$ the initial OD vector matrix for SQP algorithm optimization
*W*' the initial weight matrix in the group of all paths connecting each OD pair
*w*'_*mn*_ the initial weight element of all paths connecting nodes *m* and *n*, *m*,*n* ∈ *M*
*W* the weight matrix in the group of all paths connecting each OD pair
*w*_*mn*_ the weight element of all paths connecting nodes *m* and *n*, *m*,*n* ∈ *M*
*E* the identity matrix with the same dimension as the initial weight matrix
*G* the vector matrix of observed link flows, where *G*_*i*_ is the *i*th element of *G*
*U* the covariance matrix for the target OD vector and estimated OD vector
*V* the covariance matrix for the observed link flows and estimated link flows
*P* the matrix of link choice proportions, where *p*_*kj*_ is the *kj*th element of *P*. This is equivalent to the proportion of OD pair *j* traveling on the observed link *k*
*P*_*rj*_ the probability of path *r* that connects OD pair *j* being chosen for a trip
*x*_*k*_ the observed traffic flow of link *k*
$x_{k}^{\left(s\right)}$ the estimated traffic flow of link *k* at the *s*th iteration in the traffic assignment stage
$y_{k}^{\left(s\right)}$ the auxiliary mean traffic flow of link *k* at the *s*th iteration in the traffic assignment stage
*θ* the estimated dispersion parameter for OD estimation
$\bar{\theta }$ the target dispersion parameter for OD estimation
$\tilde{\theta }$ the initial dispersion parameter for SQP algorithm optimization
*Q* the covariance matrix for *θ* and $\bar{\theta }$
*S*_*d*_ the feasible solution set for OD matrix
*S*_*θ*_ the feasible solution set for *θ* parameter
$\sigma_{d_{j}}^{2}$ the variance for OD demands
$\sigma_{x_{k}}^{2}$ the variance for link flows
$\sigma_{\theta }^{2}$ the variance for dispersion parameter
*λ*_*d*_ random term for the target OD matrix
*λ*_*f*_ the random term for observed link flows
*F*_1_ the “distance” between the estimated OD vector matrix *d* and target demand OD matrix $\bar{d}$
*F*_2_ the “distance” between the estimated link flow vector *f* and observed link flow vector $\overset{\wedge }{f}$
*F*_3_ the “distance” between the estimated dispersion parameter *θ* and the target dispersion parameter $\bar{\theta }$
*a*_*krj*_ decision variable, if the link *k* lies on path *r* connecting the OD pair *j*, and set *a*_*krj*_ = 1, and 0 otherwise
*η* the percentage of traffic flow traveling from each node to the most adjacent node
*RMSE*(*OD*) the root mean squared error between estimated and true OD matrices
*RMSE*(*LF*) the root mean squared error based on estimated and observed (true) link flows

Based on the above notations, the OD estimation model development and validation procedure can be described as follows:

1. A ground truth OD matrix is used as prior information to calculate the link flows based on the link choice proportion model. These link flows represent the measured traffic flows obtained through fixed mechanical sensors or other means;
2. The observed link flows are chosen from those calculated link flows at fixed points throughout the network;
3. The estimated OD matrix, link flows, and dispersion parameter are obtained via the fixed-point model and two-stage iterative algorithm using the partial observed link flows from step (2);
4. Results are evaluated and compared with the ground truth as established in step (1).

#### The Fixed Point Model with Dynamic Dispersion Parameter

As presented in the previous subsection, the estimated OD vector matrix is expressed as *d* = [*d*_1_,*d*_2_,…,*d*_*j*_,…,*d*_*τ*_]*'*, where *d*_*j*_ denotes the mean traffic flow of the *j*th element of *d* for OD pair *j*. Consider an OD pair *j* connected by a link *k* which is associated with a link performance cost function *c*_*k*_(*f*_*k*_) equal to the cost of using link *k*. The link performance cost function [50] is expressed during the traffic assignment procedure in Eq 1:

$$c_{k}(f_{k})=t_{k}[1.0+\alpha_{k}{\left(\frac{f_{k}}{C_{k}}\right)}^{\beta_{k}}],\forall k\in K$$ (1)

The link flow vector is defined as *f* = [*f*_1_,*f*_2_,…,*f*_Γ_]*'*, and the matrix of link choice proportions is denoted as *P* = [*p*_*kj*_], where 0 ≤ *p*_*kj*_ ≤ 1. This represents the proportion of OD pair *j* connected by the link *k*. Thus, the mathematical expectation of link flow vector *f* can be calculated as *E*[*f*] = [*Pd*]_Γ×1_, where *Pd* is the product of the observed mean link flow vector and the matrix of link choice proportions *P*. *P* can be adjusted by the link flows and the dispersion parameter *θ*.

The OD matrix can be estimated via a fixed point formulation by considering the target OD matrix and observed link flows as follows [9, 10, 13, 14, 15, 16, 18, 19, 51]:

$$\begin{array}{l} d=\;\underset{d\in S_{d}}{\text{arg min}}[F_{1}(d,\bar{d})+F_{2}(f,\hat{f})]\\ \;=\;\underset{d\in S_{d}}{\text{arg min}}[{\left(d-\bar{d}\right)}^{T}U^{-1}(d-\bar{d})+{\left(P(d)d-\hat{f}\right)}^{T}V^{-1}(P(d)d-\hat{f})] \end{array}$$ (2)

Where:

*P*(*d*) = {*p*_*kj*_(*d*_*j*_)} is the assignment matrix, which represents the proportion of OD pair *j* using the observed link *k*;

f = *P*(*d*)*d* is the estimated link flow vector. *f* = {*f*_*k*_}, where *f*_*k*_ = Σ_*j*_ *p*_*kj*_(*d*_*j*_)*d*_*j*_.

In this study, the dispersion parameter is integrated into the objective function (Eq 2) [16, 19] as follows:

$$\left(d,\theta )=\underset{\begin{array}{l} d\in S_{d}\\ \theta \in S_{t} \end{array}}{\text{arg min}}[F_{1}(d,\bar{d})+F_{2}(f,\hat{f})+F_{3}(\theta ,\bar{\theta })\right]$$ (3)

This model can be seen as a Stochastic User Equilibrium (SUE) problem [13]. The Generalized Least Square (GLS) estimator can be used to solve Eq 3 by minimizing the Weighted Euclidean Distances (WED) between the target data and the solution vector, and Eq 3 can be then reorganized as shown in Eq 4 [9, 10, 15, 48]:

$${\left(d,\theta \right)}^{GLS}=\underset{\begin{array}{l} d\in S_{d}\\ \theta \in S_{t} \end{array}}{\text{arg min}}[{\left(d-\bar{d}\right)}^{T}U^{-1}(d-\bar{d})+{\left(P(d,\theta )d-\hat{f}\right)}^{T}V^{-1}(P(d,\theta )d-\hat{f})+{\left(\theta -\bar{\theta }\right)}^{2}Q^{-1}]$$ (4)

Where:

*P*(*d*,*θ*) = {*p*_*kj*_(*d*_*j*_,*θ*)} is the assignment matrix, and is a function of both OD matrix and dispersion parameter *θ*.

*f* = *P*(*d*,*θ*)*d* is the estimated link flow vector. *f* = {*f*_*k*_}, where *f*_*k*_ = Σ_j_ *p*_*kj*_(*d*_*j*_,*θ*)*d*_*j*_.

The matrix for link choice proportions *P* can be generally assumed fixed during the optimization procedure [10, 13, 14, 19]. This procedure performs well for uncongested traffic conditions or an idealized traffic network with fixed link costs. However, when the network becomes congested, users’ choices are increasingly influenced by adverse traffic condition. In this case, link flow and cost are not independent, and the assignment matrix *P* should be assumed to vary within each optimization step for link flow and OD estimation. Similarly, the GLS estimators of *d* and *θ* can be also obtained by solving Eq 4.

#### The Link Choice Proportion Calculation Using the Dispersion Parameter

As mentioned in notation and definitions subsection, the link flow and cost will be updated when a new set of values of *d* and *θ* is received. Drivers’ link choice decisions are influenced by the network-wide traffic condition, and thus the link choice proportion matrix *P* should be allowed to vary as well. The method of successive average (MSA) is adopted to calculate equilibrium link flows in the traffic assignment procedure [7, 16, 45, 52].

The cost of path *r* connecting the OD pair *j* can be expressed as:

$$c_{rj}=\underset{k}{\sum }a_{krj}c_{k},\;\forall k\in K$$ (5)

The probability *P*_*rj*_ can be then computed according to the path choice logit model [45]:

$$\begin{array}{ll} P_{rj} & =\;\frac{\text{exp}(-c_{rj}\theta )}{\underset{a}{\sum }\text{exp}(-c_{aj}\theta )}\\ & =\;\frac{1}{\underset{a}{\sum }\text{exp}(-c_{aj}\theta )}\text{exp}(-\theta \underset{k}{\sum }a_{krj}c_{k})\\ & =\;\frac{1}{\underset{a}{\sum }\text{exp}(-c_{aj}\theta )}[\text{exp}(-\theta a_{1rj}c_{1})\cdot \text{exp}(-\theta a_{2rj}c_{2})\cdot \ldots \cdot \text{exp}(-\theta a_{Trj}c_{T})] \end{array}$$ (6)

For a driver traveling along the path *r*, the weight assigned to link *k* is equal to exp(-*c*_*k*_*θ*). It is worth noting that the sum of probabilities over all feasible paths for each OD pair is equal to one.

As previously noted, *W*' = [*w*'_*mn*_] is the initial weight matrix of all possible paths connecting each OD pair. With the initial weight is set to *w*'_*mn*_ = exp(-*c*_*k*_*θ*), then *W*', $W_{2}^{\prime }$, and $W_{3}^{\prime }$ represent the weight matrix in the group of paths with one link, two links and three links respectively. Therefore, the weight matrix for all possible paths can be formulated as:

$$W\prime +W_{2}^{\prime }+W_{3}^{\prime }+\cdots ={\left(E-W\prime \right)}^{-1}-E$$ (7)

Wong [53] and Lo and Chan [16] have proven that the right side of Eq 7 is convergent for any acyclic networks, and is equal to *W* = (*E*−*W*')^−1^ − *E*. Therefore, the probability of a trip from node *m* to node *n* (OD pair *j*) choosing link *k* can be calculated as follows:

$$p_{kj}=\frac{w_{mg}\text{exp}(-\theta c_{k})w_{\nu n}}{w_{mn}}$$ (8)

Where link *k* connects node *g* and node *v*, and *w*_*mn*_ expresses weight matrix of all possible paths connecting nodes *m* and *n*, *m*,*n* ∈ *M*. *w*_*mn*_ is set to 1 for all nodes in the network.

Following the previous definition, the auxiliary mean traffic flow $y_{k}^{\left(s\right)}$ of link *k* is defined for each incoming *d* and *θ* via the following equation:

$$\begin{array}{ccc} y_{k}^{\left(s\right)} & = & {\left[Pd\right]}_{k}\\ & = & {\underset{}{\sum }}_{j}p_{kj}d_{j} \end{array},\;\forall k\in K$$ (9)

The equilibrium traffic link flows can be then obtained using the MSA method. Specifically, the flow of link *k* can be calculated at the (*s*+1)th iteration with the following equation:

$$\begin{array}{ccc} x_{k}^{\left(s+1\right)} & = & x_{k}^{\left(s\right)}+\frac{1}{s}(y_{k}^{\left(s\right)}-x_{k}^{\left(s\right)})\\ & = & \frac{1}{s}\sum_{j=1}^{s}y_{k}^{\left(j\right)} \end{array},\;\forall k\in K$$ (10)

As shown in Eq 10, the flow of link *k* at the (*s*+1)th iteration is equal to the mean of the auxiliary traffic flow of link *k* in the previous *s* iterations.

When a new set of values of *d* and *θ* is received, the matrix *P* of link choice proportions is updated following the procedure described above, and is then integrated into the Eq 4 to update the values of *d* and *θ*. This optimization procedure continues until convergence of the OD matrix and dispersion parameter estimation is reached.

## Model Solution Algorithm

To solve the Stochastic User Equilibrium (SUE) problem described above, a two-stage algorithm for GLS estimation and SUE traffic assignment is proposed: First, the OD matrix *d* and the dispersion parameter *θ* are simultaneously estimated under the condition of the fixed link flows, link costs, and weight matrix. Second, the link flows, link costs, and link choice proportions are updated according to the new values of *d* and *θ* in the SUE assignment process. The two-stage algorithm is executed iteratively until the convergence of values of *d* and *θ* is reached. Sequential quadratic programming (SQP) from the extended quasi-Newton method is chosen as the solution method [49].

### Two-Stage Algorithm

The initialization procedure of the two-stage algorithm can be described as follows:

1. Initialize the counter *t* = 0, set the initial OD vector matrix $d^{\left(0\right)}=\bar{d}$, the initial dispersion parameter $\theta^{\left(0\right)}=\bar{\theta }$, and the initial link flow $x_{k}^{\left(0\right)}=0$, *k* ∈ *K*.
2. Calculate the initial link costs for all links in the network using Eq 1, and calculate the weight matrix *W* for all paths based on the initial link costs and *θ*^(0)^.
3. Calculate the link choice proportion matrix *P* using the weight matrix *W* and *θ*^(0)^.
4. Calculate the initial mean auxiliary traffic flow for all the observed links with Eq 9, and update *t* = *t* + 1.

The first stage of the algorithm is described as follows:

Step 1. The objective function (Eq 4) can be updated with the new mean auxiliary observed link flows as follows:
  $${\left(d^{\left(t\right)},\theta^{\left(t\right)}\right)}^{GLS}=\underset{\begin{array}{l} d\geq 0\\ \theta >0 \end{array}}{\text{arg min}}[{\left(d^{\left(t\right)}-\bar{d}\right)}^{T}U^{-1}(d^{\left(t\right)}-\bar{d})+{\left(P^{\left(t\right)}d^{\left(t\right)}-\hat{f}\right)}^{T}V^{-1}(P^{\left(t\right)}d^{\left(t\right)}-\hat{f})+{\left(\theta^{\left(t\right)}-\bar{\theta }\right)}^{2}Q^{-1}]$$ (11)
  Where:
  *U*^−1^, *P*^(t)^, *V*^−1^, and *Q*^−1^ can be updated using the new mean auxiliary observed link flows, estimated OD vector matrix, dispersion parameter, and link flow vector respectively;
  The feasible set for *d* and *θ* should meet the requirements d ≥ 0, *θ > 0*. When the value of *θ* approaches zero, the path choice probabilities for all paths tend to be equal. As the value of *θ* increases, the path choice probabilities tend to be deterministic.
Step 2. Use the SQP algorithm to obtain a new set of values of *d*^(t)^ and *θ*^(t)^ that minimizes the objective function. The starting point for optimizing the OD vector ${\tilde{d}}^{\left(t\right)}$ and dispersion parameter ${\tilde{\theta }}^{\left(t\right)}$ should be fixed in advance. During the iterative process of the SQP algorithm, whenever a new value *θ* is received, the link choice proportion matrix *P* will be updated by changing the value of exp(−*θc*_*k*_) in Eq 8, while the link cost and weight matrix should remain unchanged.
  The second stage of the algorithm can be described as follows:
Step 3. Initialize the counter *s* = 1.
Step 4. Calculate the weight matrix *W* with the new dispersion parameter *θ*^(*t*)^.
Step 5. Calculate the link choice proportion matrix *P*^(*t*)^ using the weight matrix *W* and dispersion parameter *θ*^(t)^.
Step 6. Calculate the mean auxiliary traffic flow for all observed links as follows:
  $$y_{l}^{\left(s\right)}={\left[P^{\left(t\right)}d^{\left(t\right)}\right]}_{l}=\sum_{j}p_{lj}d_{j},\;l\in L$$
Step 7. Calculate the equilibrium traffic link flow of link *k* via the MSA method:
  $$x_{l}^{\left(s+1\right)}=x_{l}^{\left(s\right)}+\frac{1}{s}(y_{l}^{\left(s\right)}-x_{l}^{\left(s\right)}),\;l\in L$$
Step 8. The maximum relative difference between current and previous mean link flows should satisfy the following requirement:
  $$\underset{\forall l\in L}{\text{max}}\left\{\frac{\left|x_{l}^{\left(s+1\right)}-x_{l}^{\left(s\right)}\right|}{x_{l}^{\left(s+1\right)}}\right\}\leq \varepsilon_{1}$$ (12)
  If the above requirement is met, the algorithm proceeds directly to step 11, otherwise proceed to step 9.
Step 9. Calculate the new link costs according to $x_{l}^{\left(s+1\right)}$, *l* ∈ *L*.
Step 10. Calculate the weight matrix using the updated link costs, set *s* = *s* + 1, and return to step 5.
Step 11. The maximum relative difference between the current and previous OD matrix estimates should satisfy the following requirement:
  $$\underset{\forall j\in J}{\text{max}}\left\{\frac{\left|d_{j}^{\left(t\right)}-d_{j}^{\left(t-1\right)}\right|}{d_{j}^{\left(t\right)}}\right\}\leq \varepsilon_{2}$$ (13)
  If the above requirement is met, terminate the procedure and output the current estimates of OD vector matrix *d* and dispersion parameter *θ* as *d*^(*t*)^ and *θ*^(*t*)^. Otherwise, set *t* = *t* + 1, and proceed to step 12.
Step 12. Calculate the new starting points as follows: ${\tilde{d}}^{\left(t+1\right)}=\frac{1}{t}d^{\left(t\right)}+\frac{t-1}{t}d^{\left(t-1\right)}$, ${\tilde{\theta }}^{\left(t+1\right)}=\frac{1}{t}{\tilde{\theta }}^{\left(t\right)}+\frac{t-1}{t}{\tilde{\theta }}^{\left(t-1\right)}$, and return to step 1.

### Model Evaluation

To evaluate the performance of the proposed method, the root mean squared errors (RMSE) for OD matrix and link flows after convergence are defined as follows:

(1) The root mean squared error (RMSE) of the estimated link flows $x_{l}^{\left(s+1\right)}$ relative to the true link flow *x*_*l*_ is computed as follows:
  $$RMSE(LF)=\sqrt{\frac{1}{\Gamma }\overset{\Gamma }{\underset{l=1}{\sum }}{\left(x_{l}^{\left(s+1\right)}-x_{l}\right)}^{2}}$$ (14)
  Similarly, the RMSE of the observed (target) link flows ${\hat{f}}_{l}$ relative to the true link flows *x*_*l*_ can be defined as RMSE $\left(L\bar{F}\right)$, where $x_{l}^{\left(s+1\right)}$ is replaced by ${\hat{f}}_{l}$ in Eq 14.
(2) The RMSE of the estimated OD matrix *d*^(t)^ relative to the true OD matrix *d* can be defined as RMSE (OD):
  $$RMSE(OD)=\sqrt{\frac{1}{\tau }\overset{\tau }{\underset{j=1}{\sum }}{\left(d_{j}^{\left(t\right)}-d_{j}\right)}^{2}}$$ (15)
  Likewise, the RMSE of target OD matrix $\bar{d}$ relative to the true OD matrix *d* is defined as RMSE ($O\bar{D}$), where *d*^(t)^ is replaced by ${\bar{d}}_{j}$ in Eq 15.

## Numerical Experiment and Result Analysis

### A Hypothetical Network Test

**Fig 1:**
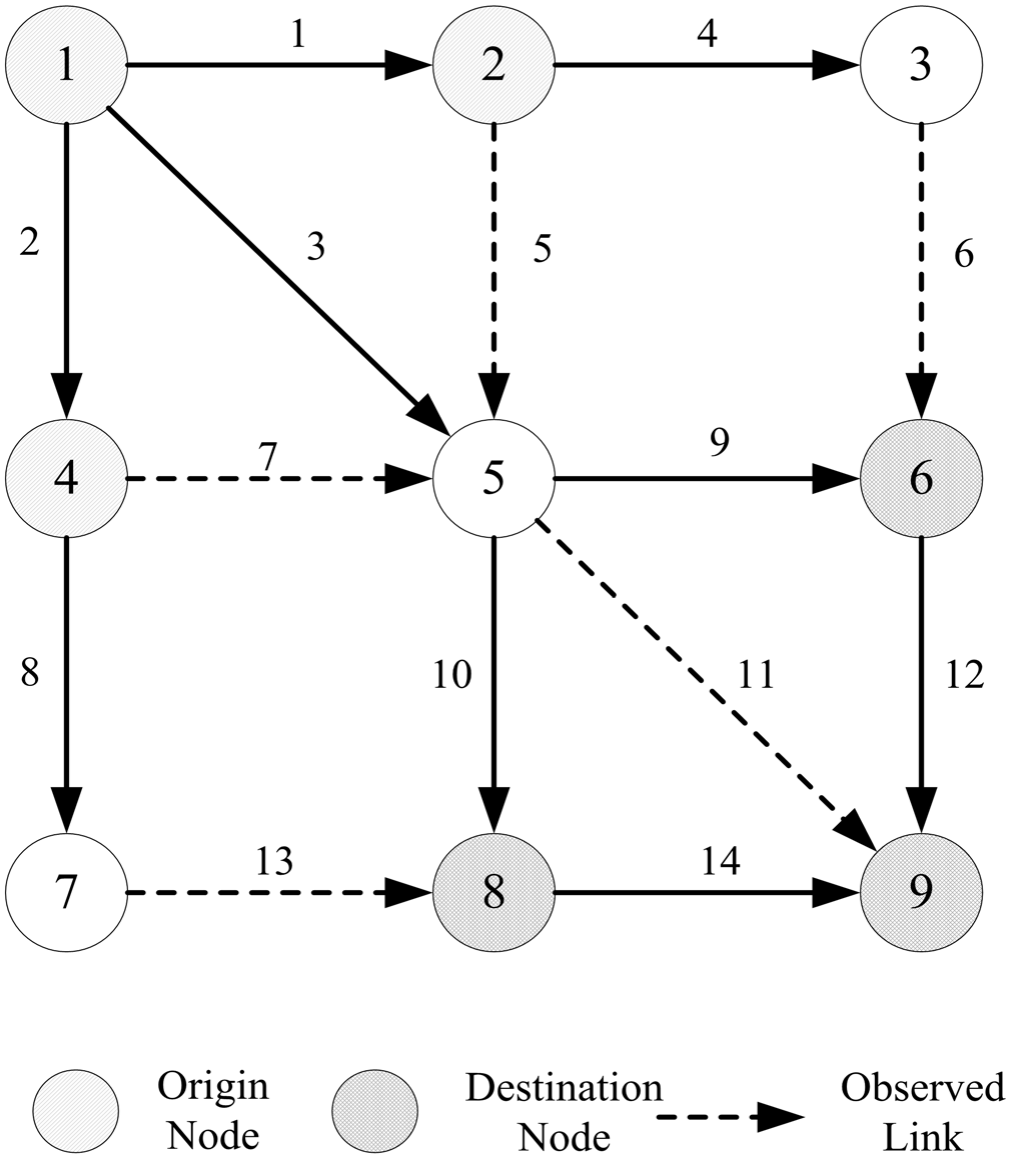
The test network used in the numerical example.

In this section, the performance of the proposed approach is tested in a hypothetical network. The network and data proposed by Yang et al. [15] and Caggiani et al. [19] are adopted as the test bed with some slight modifications. The network (presented in Fig 1), is composed of 9 nodes (3 origin centroids and 3 destination centroids), and 14 links. The true and initial OD vector matrices *d* and $\tilde{d}$ for the SQP algorithm are shown in Table 1. The initial dispersion parameter $\tilde{\theta }$ is assumed to be 4, and the true dispersion parameter $\hat{\theta }$ is fixed to 1.5. Note that the initial OD matrix $\tilde{d}$ and dispersion parameter $\tilde{\theta }$ are quite dissimilar from those of the ground truth data.

**Table 1:** The true and initial OD vector matrices.

OD pair	1–6	1–8	1–9	2–6	2–8	2–9	4–6	4–8	4–9
*j*	1	2	3	4	5	6	7	8	9
*d*	120	150	100	130	200	90	80	180	110
$\tilde{d}$	30	20	10	30	30	30	30	40	20

The following parameters in the Bureau of Public Roads (BPR) [50] link performance function are used: *α*_*k*_ = 0.15 and *β*_*k*_ = 4, ∀*k* ∈ *K*. In addition, the free flow travel time (*t*_*k*_) and capacity (*C*_*k*_) for each link are predetermined as shown in Table 2.

**Table 2:** Free flow travel time and capacity for each link.

link	1	2	3	4	5	6	7	8	9	10	11	12	13	14
*t*_*k*_	2.0	1.5	3.0	1.0	1.0	2.0	2.0	1.0	1.0	1.0	2.0	1.0	1.0	1.0
*C*_*k*_	280	290	280	280	600	300	500	400	500	700	250	300	350	520

The ground truth link flows can be generated by allocating the true OD matrix to the traffic network using SUE-Logit assignment method presented in Section 2.3. The true dispersion parameter is *θ* = 1.5, resulting in the link flows shown in Table 3. The set of links {5, 6, 7, 11, 13} is selected as the observed links.

**Table 3:** True link flows in the hypothetical network.

link	1	2	3	4	5	6	7	8	9	10	11	12	13	14
*x*_*k*_	125	143	103	172	474	172	201	313	307	393	279	148	313	475

In this example, we assume that the OD vector and link flow vector follow the Poisson distribution. The covariance matrices *U* (for OD demands) and *V* (for link flows) in Eq 4 can be assumed to be diagonal matrices [9, 14, 54]. The diagonal element for *U*, *V* and *Q* can be computed respectively through the following equations:

$$\sigma_{d_{j}}^{2}={\left(cv_{d}\cdot d_{j}\right)}^{2},\;\sigma_{x_{k}}^{2}={\left(cv_{x}\cdot x_{k}^{\left(s+1\right)}\right)}^{2},\;\sigma_{\theta }^{2}={\left(cv_{\theta }\cdot \theta \right)}^{2}$$

Where *cv*_*d*_, *cv*_*x*_ and *cv*_*θ*_ represent the variation coefficients for OD demands, link flows, and dispersion parameter respectively. Specifically, these parameters are set as *cv*_*d*_ = 0.3, *cv*_*x*_ = 0.05, and *cv*_*θ*_ = 0.1.

**Fig 2:**
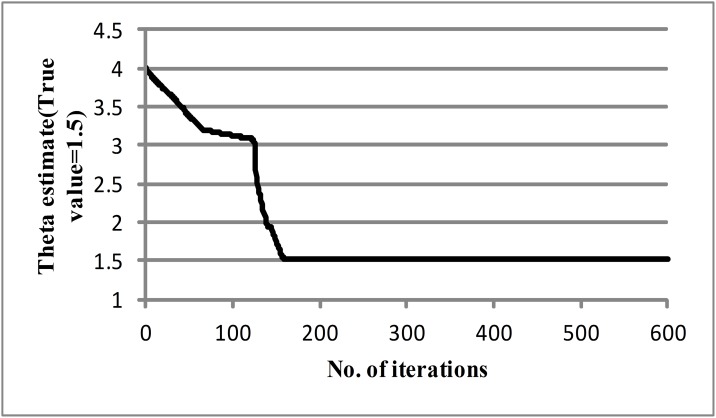
Convergence of the theta estimate.

**Fig 3:**
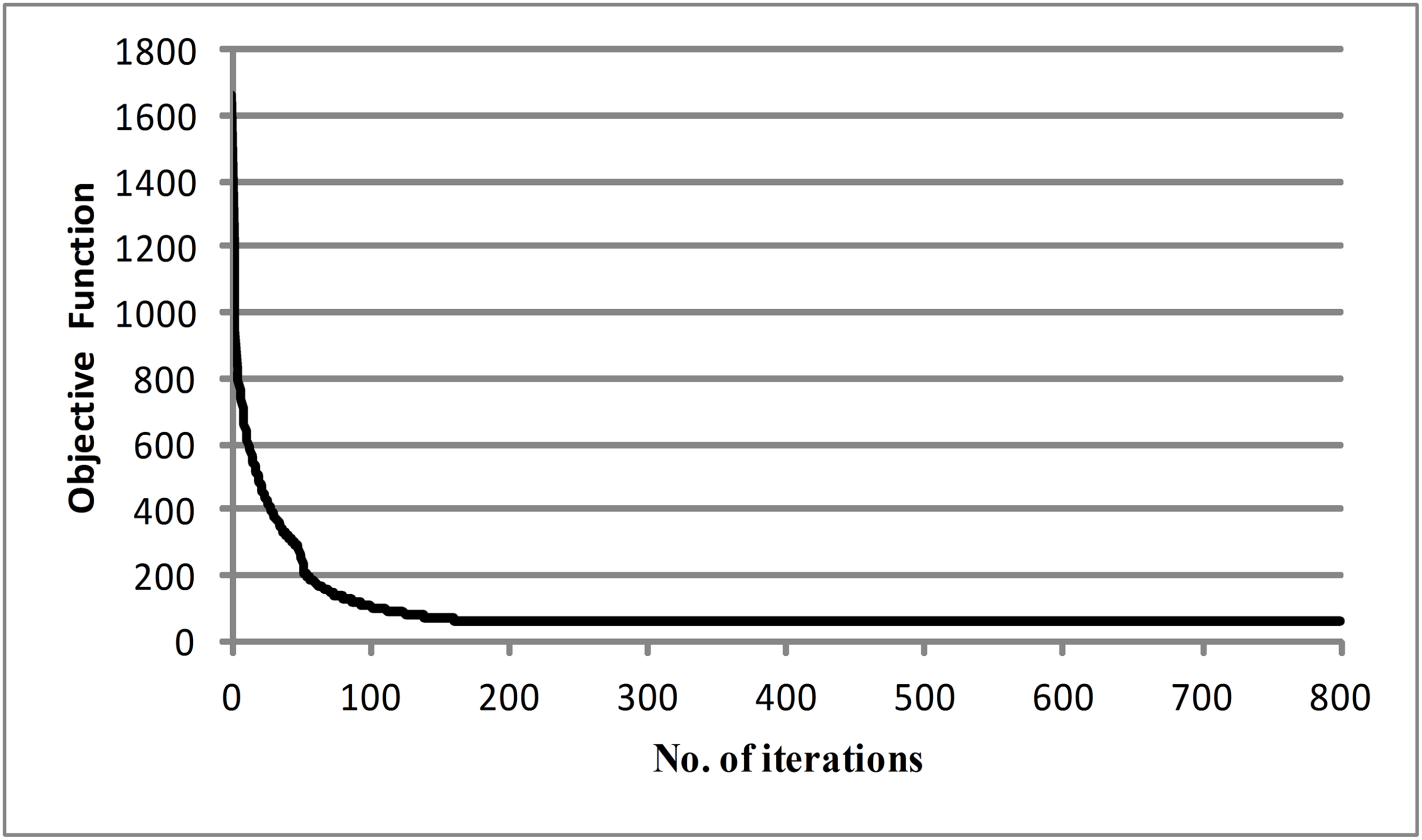
Convergence of the objective function.

The target OD matrix $\bar{d}$, observed link flow vectors $\hat{f}$, and target dispersion parameter $\bar{\theta }$ can be generated separately by adding random terms into the corresponding true values. The random terms are sampled from independent normal variables with zero means. For instance, the target OD matrix can be calculated by adding a random term with *λ*_*d*_ = 0.3 to the values of the true OD matrix divided by two, the observed link flow vectors can be generated by adding a random term with *λ*_*f*_ = 0.1, and the target parameter can be set as $\bar{\theta }\;=\;4$. In addition, the error tolerance threshold used in the optimization is set to *ε*_1_ = *ε*_2_ = 10^−3^. The convergence for theta is plotted in Fig 2, which shows the estimate slowly falling in the first 120 iterations before rapidly converging to the true value at 1.5099. This is a very slight deviation with the true value of 1.5. In addition, the convergence of the objective function is presented in Fig 3, where the value of the objective function sharply falls at the first iteration and then gradually decreases and levels off at a lower value. Poor initial choices of OD input vector and dispersion parameter may lead to the slower convergence.

**Fig 4:**
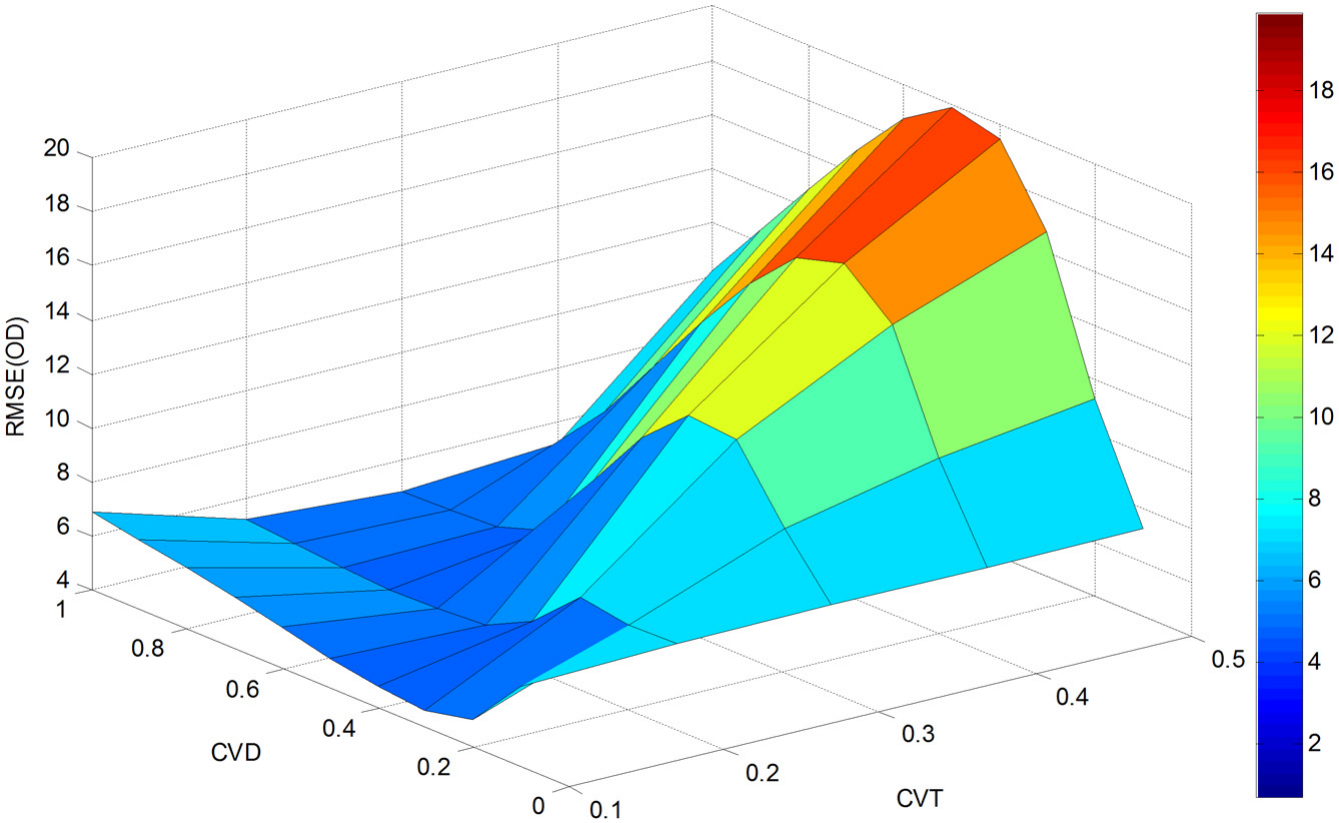
RMSE(OD) versus *cv*_*d*_ and *cv*_*θ*_.

**Fig 5:**
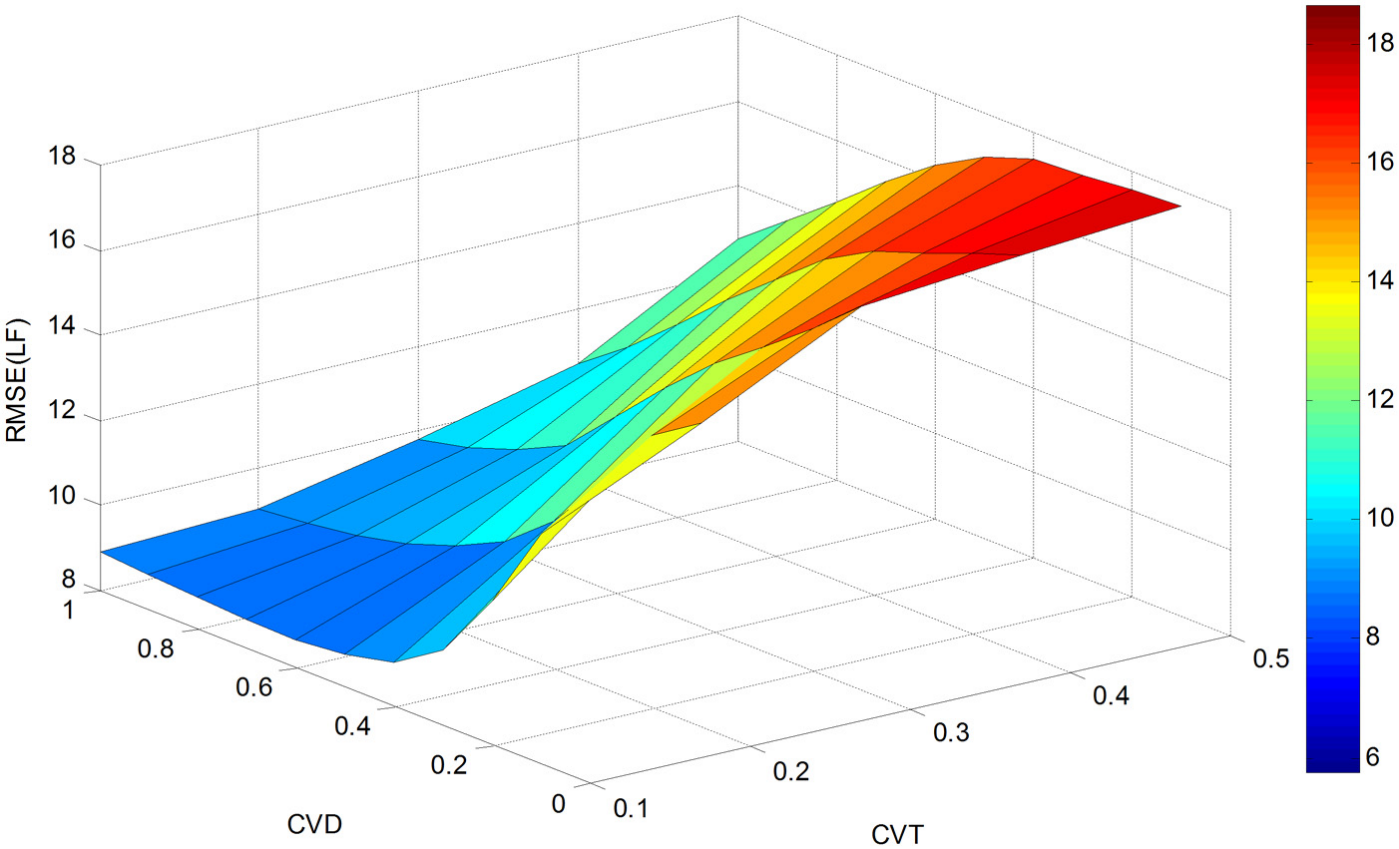
RMSE(LF) versus *cv*_*d*_ and *cv*_*θ*_.

**Fig 6:**
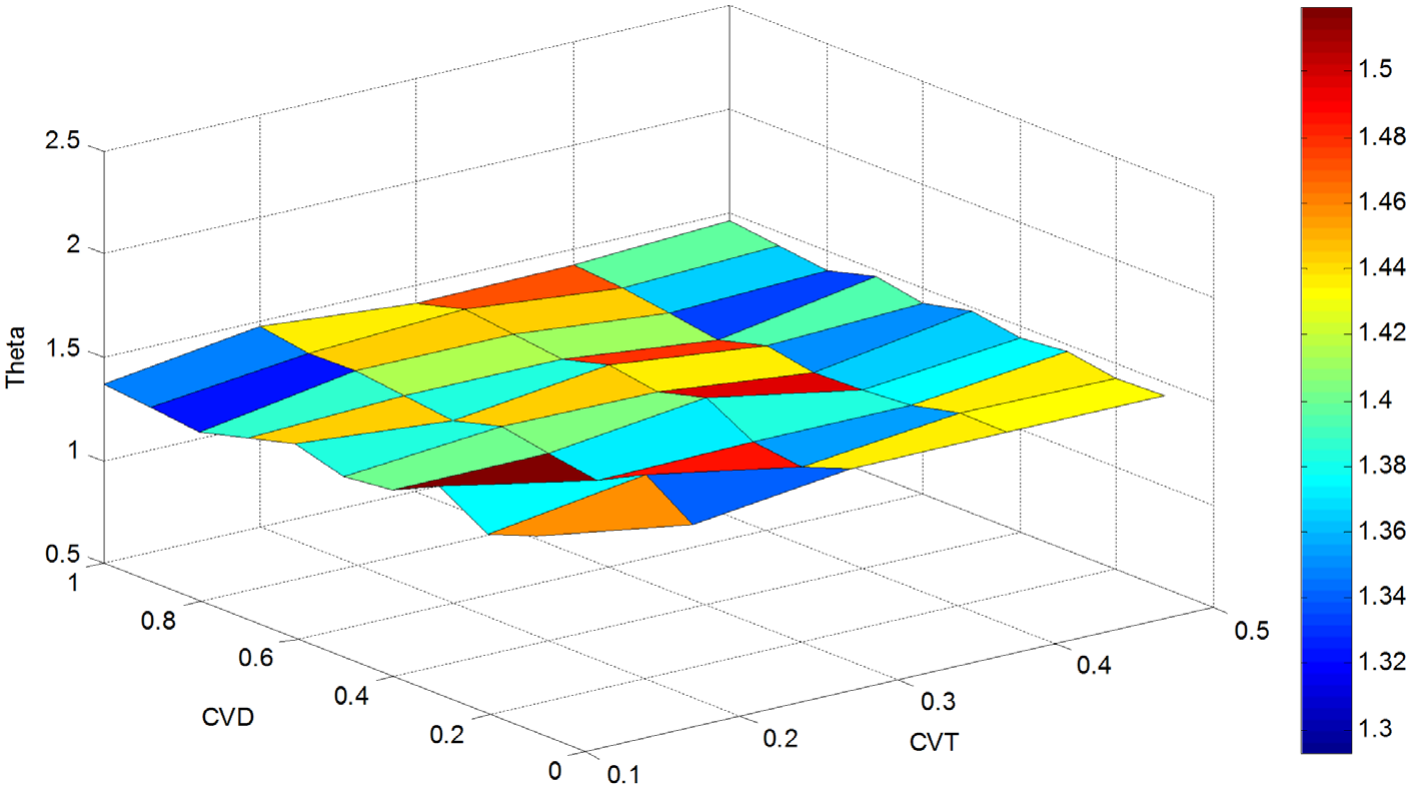
Estimated Theta versus *cv*_*d*_ and *cv*_*θ*_.

In order to further evaluate the effectiveness of the proposed approach, a sensitivity analysis is conducted with parameter *cv*_*θ*_(CVT) varying from 0.1 to 0.5 and *cv*_*d*_(CVD) changing from 0.1 to 1. This generates 50 different estimates for RMSE (OD), RMSE (LF) and Theta as presented in Figs 4–6.

As shown in Fig 4, RMSE (OD) increases with the variation coefficient *cv*_*θ*_ when *cv*_*d*_ falls between 0.2 and 0.8. With *cv*_*θ*_ fixed between 0.3 and 0.5, RMSE(OD) can be seen as a convex function of *cv*_*d*_. Alternatively, when *cv*_*θ*_ is between 0.1 and 0.2, *cv*_*d*_ has a negligible impact on RMSE (OD). Thus, the maximum value (19.9022) of RMSE (OD) can be found at *cv*_*d*_ = 0.5 and *cv*_*θ*_ = 0.5, and the minimum value (4.6859) is obtained at *cv*_*d*_ = 0.3 and *cv*_*θ*_ = 0.1. Compared with the initial RMSE (OD) of 93.8971 calculated from Table 1, a 78.8% reduction is achieved at the maximum RMSE (OD), and a 95% reduction is obtained at the minimum RMSE (OD).

Fig 5 shows the impact of *cv*_*d*_ and *cv*_*θ*_ on RMSE (LF). With the value of *cv*_*d*_ fixed, RMSE (LF) increases with the variation coefficient *cv*_*θ*_. For a fixed value of *cv*_*θ*_, the RMSE (LF) decreases with an increase in *cv*_*d*_. Thus, we can conclude that maximum RMSE (LF) value of 19.6597 is located at *cv*_*d*_ = 0.1 and *cv*_*θ*_ = 0.5, and the minimum value of 8.6696 can be found at *cv*_*d*_ = 0.6 and *cv*_*θ*_ = 0.1. Compared with the initial RMSE (LF) value of 30.8347, 36.2% and 71.9% reductions can be achieved for the maximum value of RMSE (LF) and minimum value of RMSE (LF) respectively.

As shown in Fig 6, the value of theta varies negligibly with the choice of *cv*_*d*_ and *cv*_*θ*_. In other words, the estimated value of theta always converges to approximately the true value. As shown in Fig 6, for a fixed value of *cv*_*d*_, the estimated *θ* is close to the true value for any given *cv*_*θ*_. For example, the value of *θ* fluctuates between 1.37 and 1.51 when *cv*_*d*_ = 0.3. Likewise, for any fixed *cv*_*θ*_, the estimated *θ* varies minimally about the true value of *θ* using the proposed method. For example, the estimated *θ* is between 1.35 and 1.52 for *cv*_*θ*_ = 0.1.

The above discussion reveals a fact that the initial value of $\bar{d}$, $\bar{\theta }$, and observed link flow vectors $\hat{f}$ do not affect the theta estimation performance. This is equivalent to a convex optimization problem, where the optimal results tend to converge near the true dispersion parameter value. This implies that the estimate of *θ* is insensitive to the variation coefficients, and can be used as a stable and accurate parameter to determine travelers’ route decisions.

### Comparison and Analysis

To further demonstrate the advantages of the proposed methodology, two OD matrix estimation methods are implemented and compared with the proposed approach. To make this comparison, we first implement the algorithm described in Yang et al. [15], which presents an optimization model for OD matrix estimation in congested networks using the logit-based SUE. The method described in Lo and Chan [16] is implemented for the second comparison. This method applies both statistical estimation and traffic assignment to simultaneously calculate the OD matrix and link choice proportions based on OD survey data and traffic counts. To maintain a fair comparison, the same test network and data set are applied in all cases.

The OD matrix estimation method proposed by Yang et al. [15] is given in section 2.2. The objective function is shown in Eq 16.

$$\begin{array}{ccc} \left(d,\theta \right) & = & \underset{d\in S_{d}}{\text{arg min}}[F_{1}(d,\bar{d})+F_{2}(f,\hat{f})]\\ & = & \underset{\begin{array}{l} d\geq 0\\ \theta >0 \end{array}}{\text{arg min}}[\frac{1}{2}{\left(d-\bar{d}\right)}^{2}+\frac{1}{2}{\left(Pd-\hat{f}\right)}^{2}] \end{array}$$ (16)

In Yang et al.’s work, the weighted Euclidean distance function is used to develop a unit weighting matrix and the value of theta is set to 1.5. The RMSE(OD), RMSE(LF), RMSE $\left(O\bar{D}\right)$ and RMSE($\left(L\bar{F}\right)$ for Yang et al.’s approach are calculated and compared with the proposed approach in Table 4:

**Table 4:** Comparison between Yang et al.’s approach and the proposed approach with OD matrix and link flow estimation.

Approach	Estimated OD matrix		Estimated Link flows	
	RMSE $\left(O\bar{D}\right)$	RMSE (OD)	RMSE $\left(L\bar{F}\right)$	RMSE (LF)
Yang et al.’s approach	24.27	20.85	26.65	19.02
Proposed approach	24.27	18.79	26.65	17.39

As shown in Table 4, the proposed method yields significantly lower RMSE (OD) and RMSE (LF) relative to Yang et al.’s approach. Compared with the initial RMSE values, a 22.6% reduction in RMSE (OD) is achieved using the proposed approach, while only a 14.1% reduction is achieved using the method described in Yang et al. Similarly, the proposed approach resulted in a 34.7% reduction in RMSE(LF), while only a 28.6% reduction was achieved using Yang et al.’s approach. One reason that the dispersion parameter is estimated and integrated into the Eq 3 by $F_{3}(\theta ,\bar{\theta })$ in the proposed method, and it is able to yield a better estimate of the dispersion parameter than previous approaches. The other reason is that the covariance matrices *U* (for OD demands), *V* (for link flows) and *Q* (for dispersion parameter) are not a fixed variable during the calculation. These improvements can help the method enhance the estimation performance for the OD matrix and link flow vectors.

Lo and Chan [16] present the following maximum likelihood objective function:

$$\left(d,\theta )=\text{arg}{\text{max}}_{d\geq 0,\theta >0}\text{ln}L(\theta ,d|\hat{f},\bar{d}\right)$$ (17)

In Lo and Chan [16], it is assumed that the observed flows are equal to the true flows in the test network. For Lo and Chan’s algorithm, we set the target dispersion parameter to $\bar{\theta }=4$ (This is also equal to the initial dispersion parameter value used in Lo and Chan [16]’s work), and the variation coefficients as follows: *cv*_*θ*_ = 0.1, *cv*_*x*_ = 0.05, and *cv*_*d*_ = 0.3. In order to evaluate the performance of the proposed approach relative to that of Lo and Chan [16]’s method, RMSE (OD), RMSE (LF), and the estimated Theta are selected for comparison and shown in Table 5.

**Table 5:** Comparison between Lo and Chan’s approach and the proposed approach with OD matrix, link flow and Theta estimation.

Approach	Estimated OD matrix		Estimated Theta	
	RMSE (OD)	RMSE (LF)	Theta target	Theta estimated
Lo and Chan’s approach	5.34	12.08	4	1.572
Proposed approach	4.69	9.77	4	1.509

Unlike Lo and Chan’s method, random terms are added to the observed link flows in the proposed approach, thus introducing additional challenges for estimation. However, the results presented in Table 5 demonstrate that the method proposed in this paper outperforms Lo and Chan’s approach in terms of OD matrix, link flow, and Theta estimation accuracy.

### Application to A Square Network in Seattle

**Fig 7:**
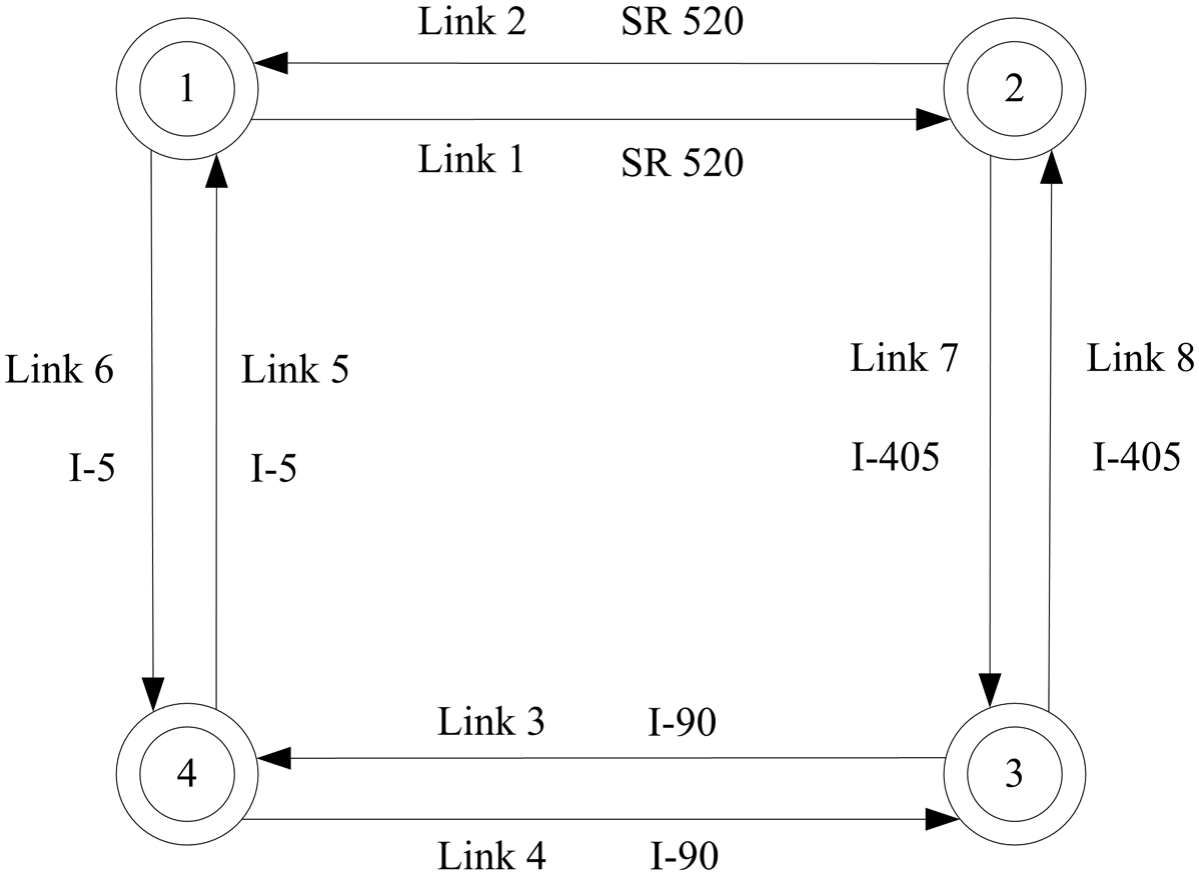
Square network in Seattle. Double circle nodes represent zone centroids (origins and destinations).

A square network in Seattle is used as a congested network case study to demonstrate the applicability and transferability of the proposed approach in a real-world traffic network (Shown in Fig 7). Empirical data was collected from loop detectors located along one freeway section in Seattle area, and obtained for this research through the Strategic Highway Research Program 2 (SHRP 2 program) supported by Washington State Department of Transportation (WSDOT) [55].

The square test network used in this case study consists of 4 nodes and 8 links, where all nodes are centroids (origins and destinations). The topology of the test network is outlined in Fig 7. We assume that the study network is acyclic, such that the traffic flow starting from one node will leave the network before returning to the original node. Specifically, Links 1 and 2 represent the SR 520 Bridge connecting I-5 in Seattle and SR 202 in Redmond. Interstate 90 (I-90) is represented by Links 3 and 4, and Interstate 5 (I-5) is represented by Links 5 and 6. Links 7 and 8 represent Interstate 405 (I-405), which intersects I-90 in the south and SR 520 in the north.

**Fig 8:**
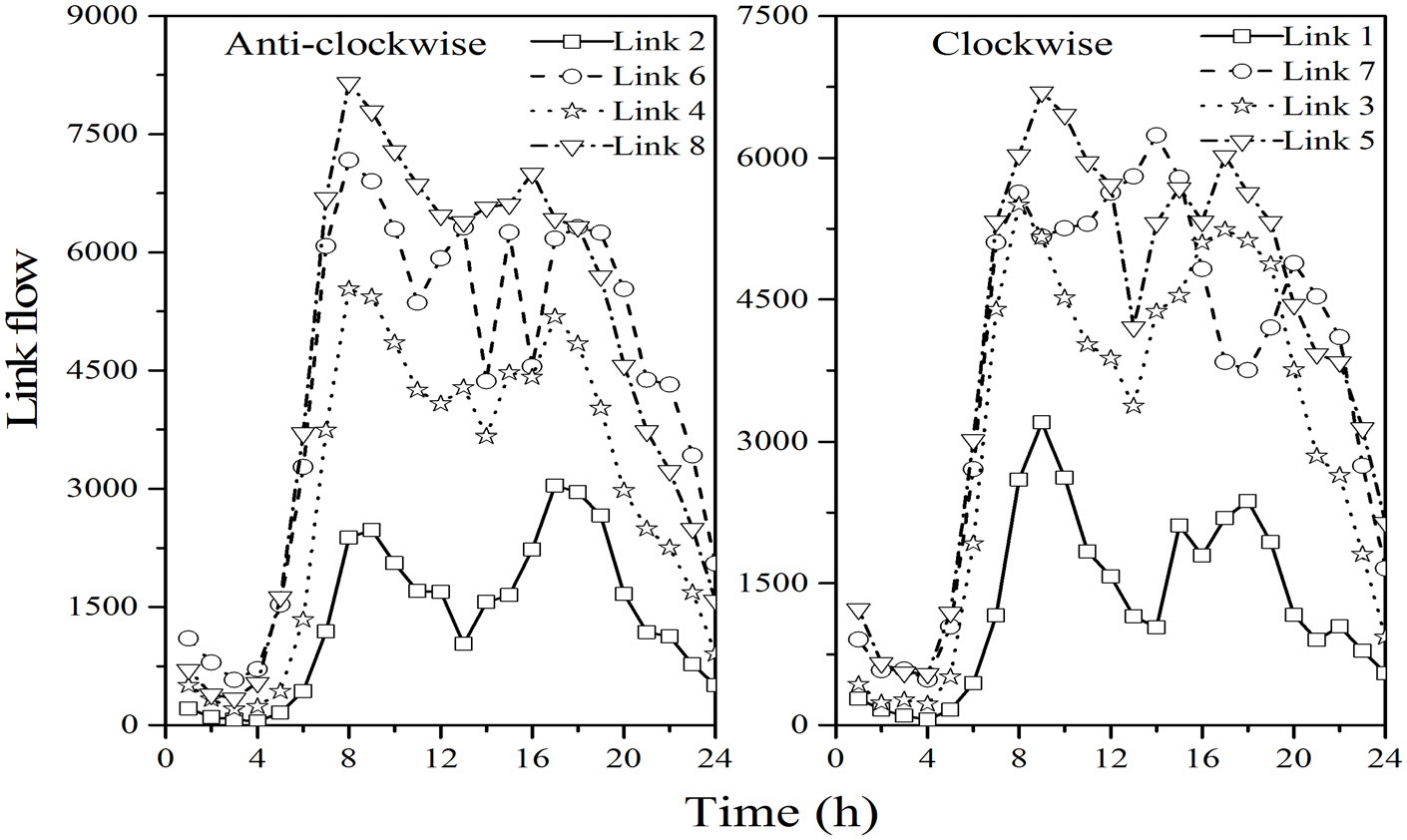
Traffic flow fluctuation by time of day.

Traffic flows were obtained from loop detectors installed at nodes 1, 2, 3 and 4, illustrated in Fig 8. The parameters for the BPR link performance cost function (Eq 18) were estimated based on the empirical data and are presented in Table 6.

$$c_{k}(f_{k})=t_{k}[1.0+\alpha_{k}{\left(\frac{f_{k}}{C_{k}}\right)}^{\beta_{k}}],\;\forall k\in K$$ (18)

**Table 6:** BPR link performance cost function parameter calibration.

Links	Road Name	Length(km)	*t*_*k*_(h)	*C*_*k*_(pcu/h)	*α*_*k*_	*β*_*k*_
1,2	SR 520	7.5	0.1162	4149	0.1450	3.5
3,4	I-90	3.6	0.0667	8685	0.1035	2.7
5,6	I-5	3.5	0.1016	9683	0.0988	2.7
7,8	I405	7.8	0.1332	7961	0.1242	3.5

Table 7 indicates the external traffic flow recorded for each node during peak hour, where 1-Link 1 represents the external traffic flow on Link 1 from node 1, and 2-Link 7 represents the external traffic flow on Link 7 from node 2, and so forth. To convert true link flows into a ground truth OD matrix, the flow proportion for each node *η* = 0.6 is assumed based on extensive video records and filed surveys. This implies that, for the traffic leaving each node, 60% exits the network from an adjacent node while 40% exits from the other nodes. In order to avoid circular flow in the OD calculation process, it is assumed that the final remaining traffic flow will leave from the last node before returning to the original node. Based on these assumptions, the ground truth OD matrix is calculated and shown in Table 8. In addition, the initial OD matrix $\tilde{d}$ can be computed by rounding the last digit of the true OD matrix as shown in Table 8.

**Table 7:** The external true traffic flow for each node at peak hour.

Link direction	1-Link 1	2-Link 2	3-Link 3	4-Link 4	4-Link 5	1-Link 6	2-Link 7	3-Link 8
Traffic flow	3199	2480	5499	5535	6018	7169	5628	8153

**Table 8:** True OD matrix and initial OD matrix at peak hour for each OD pair.

OD pair	1–2	1–3	1–4	2–1	2–3	2–4	3–1	3–2	3–4	4–1	4–2	4–3
*j*	1	2	3	4	5	6	7	8	9	10	11	12
*d*	3067	2489	4814	2389	3774	1946	3277	5772	4604	4497	2773	4284
$\tilde{d}$	307	249	482	239	378	195	328	578	461	450	278	429

The true OD matrix in Table 8 is then used to assign the corresponding traffic flow into each link according to Eq 5 through Eq 10. The calculated traffic flows can be assumed to represent the true link flows, where link 1, 3, 5, 6, and 8 are selected as the observed links to estimate OD matrix shown in Table 9.

**Table 9:** Observed link flows at peak hour.

Link No.	1	2	3	4	5	6	7	8
*x*_*k*_	3711	-	8282	-	8439	7857	-	7591

**Fig 9:**
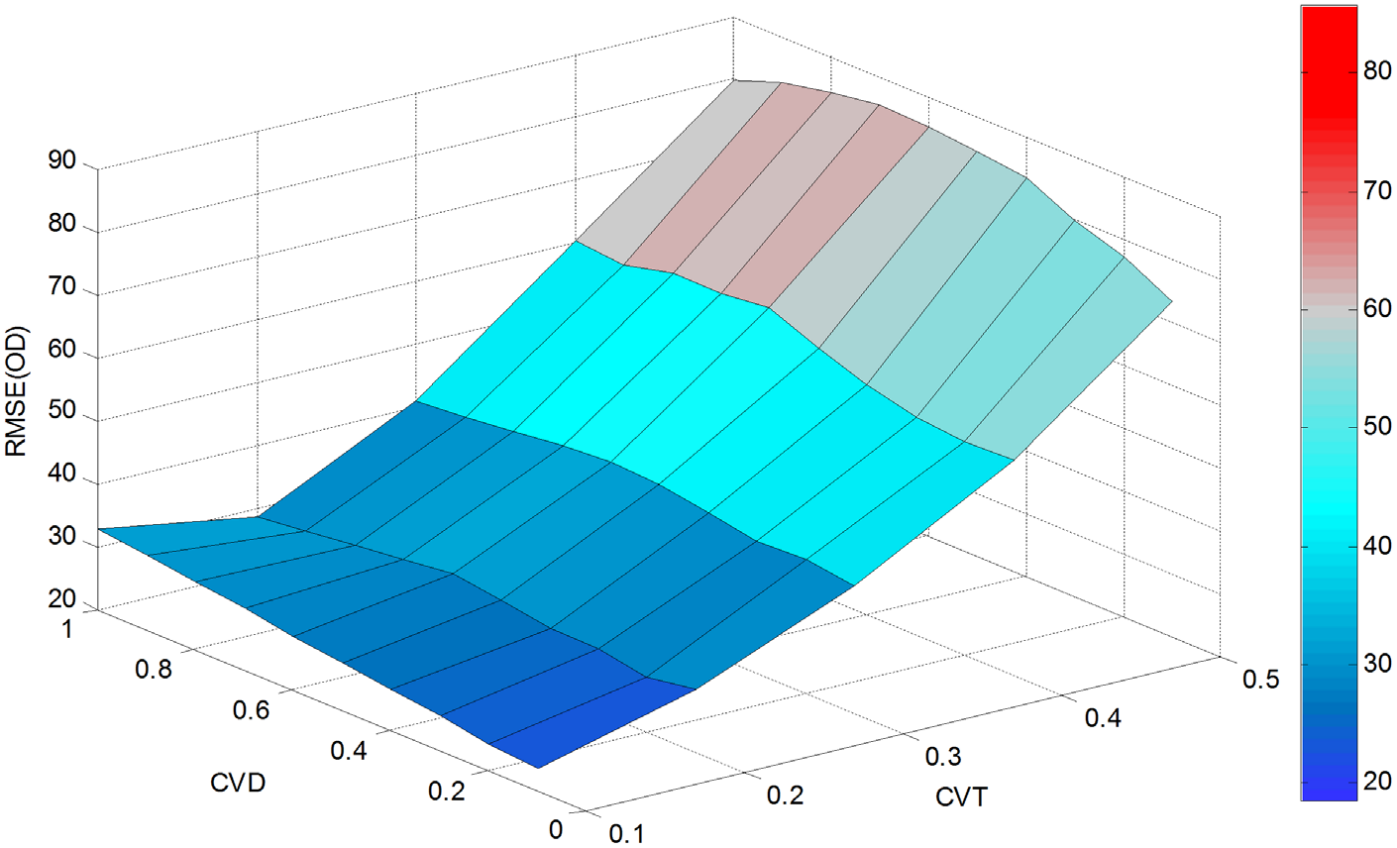
RMSE(OD) versus *cv*_*d*_ and *cv*_*θ*_ in the actual network.

**Fig 10:**
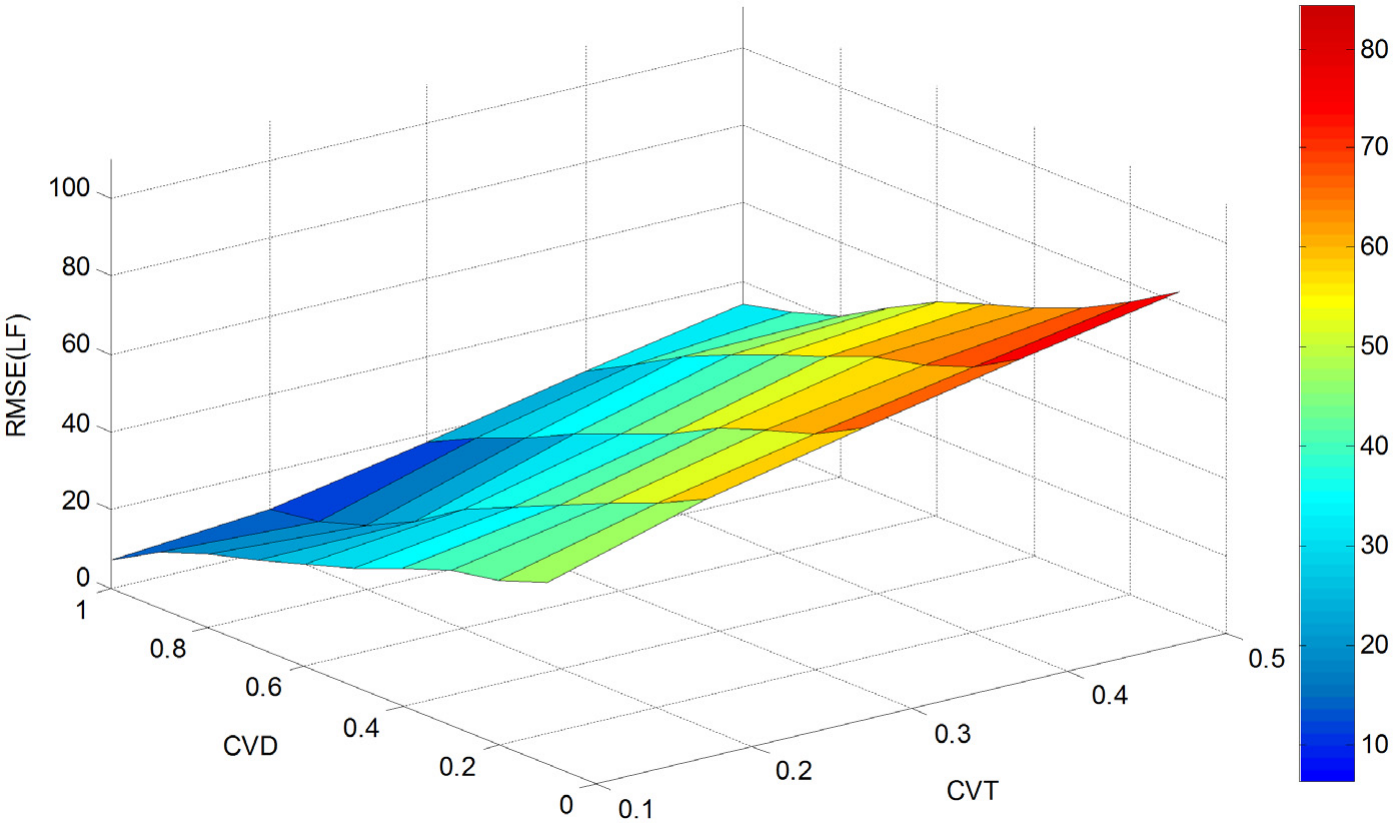
RMSE(LF) versus *cv*_*d*_ and *cv*_*θ*_ in the actual network.

**Fig 11:**
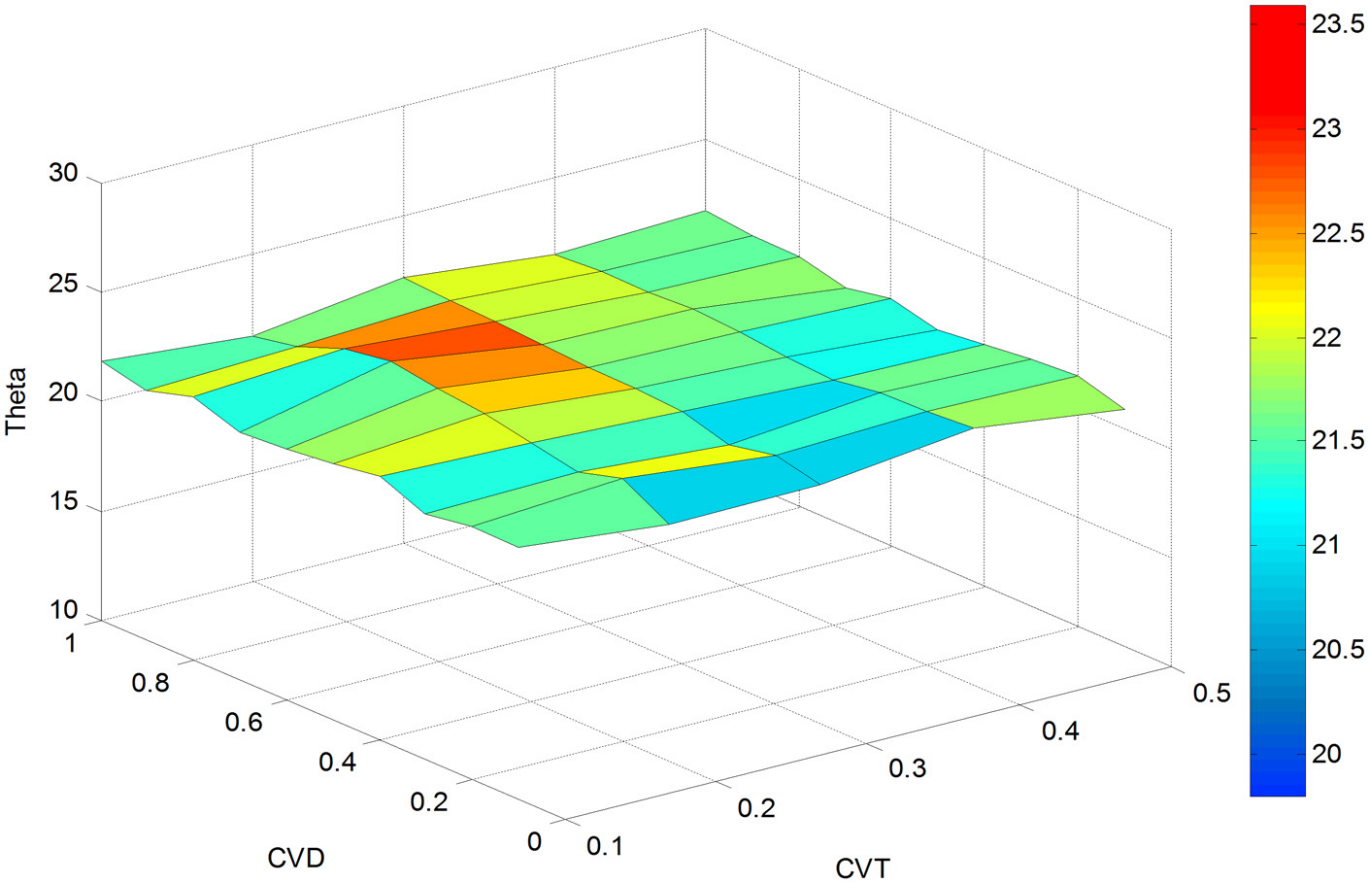
Theta estimated versus *cv*_*d*_ and *cv*_*θ*_ in the actual network.

Similar to the hypothetical network, we assume that the OD demands and observed link flows follow the Poisson distribution, and the covariance matrices *U* and *V* can be assumed to be diagonal. The initial value of the dispersion parameter $\tilde{\theta }$ is set to 40.5. The remaining input parameters are set identically to the hypothetical network. In addition, a sensitivity analysis with 50 different combinations of variation coefficients *cv*_*d*_ and *cv*_*θ*_ was conducted to investigate the optimal parameter initialization for the proposed approach. The results of this sensitivity analysis are shown in Figs 9–11.

Fig 9 shows the value of RSME (OD) versus *cv*_*d*_ and *cv*_*θ*_. For a fixed value of *cv*_*d*_ between 0.1 and 0.7, RMSE(OD) increases with *cv*_*θ*_. RMSE(OD) is a concave function of *cv*_*θ*_ when *cv*_*d*_ is fixed between 0.8 and 1.0, and a convex function of *cv*_*d*_ for a fixed value of *cv*_*θ*_ between 0.1 and 0.5. Thus, the maximum RMSE (OD) of 85.6123 can be obtained at *cv*_*d*_ = 0.7 and *cv*_*θ*_ = 0.5, and the minimum value of 23.5917 can be obtained at *cv*_*d*_ = 0.1 and *cv*_*θ*_ = 0.1.

As shown in Fig 10, the value of RMSE (LF) increases with *cv*_*θ*_ for a fixed value *cv*_*d*_. For a fixed value of *cv*_*θ*_, the value of RMSE (LF) decreases with an increase of *cv*_*d*_. The maximum RMSE (LF) of 82.5113 is found at *cv*_*d*_ = 0.1 and *cv*_*θ*_ = 0.5, and the minimum value of 7.3277 at *cv*_*d*_ = 1.0 and *cv*_*θ*_ = 0.1.

As noted in the hypothetical case, the choice of *cv*_*d*_ and *cv*_*θ*_ has very little impact on the estimation of Theta. As shown in Fig 11, the estimated dispersion parameter *θ* is between 20.8327 (*cv*_*d*_ = 0.5 and *cv*_*θ*_ = 0.5) and 22.7165 (*cv*_*d*_ = 0.7 and *cv*_*θ*_ = 0.2) in all cases. The best estimate of dispersion parameter *θ* can be found between 20.8327 and 22.7165.

Finally, using the BPR link performance cost function parameters described in Table 6, different combinations of variation coefficient *cv*_*d*_ = 0.3 and *cv*_*θ*_ = 0.1; *cv*_*d*_ = 0.5 and *cv*_*θ*_ = 0.5;*cv*_*d*_ = 0.7 and *cv*_*θ*_ = 0.2 are used to estimate theta for the actual network.

**Fig 12:**
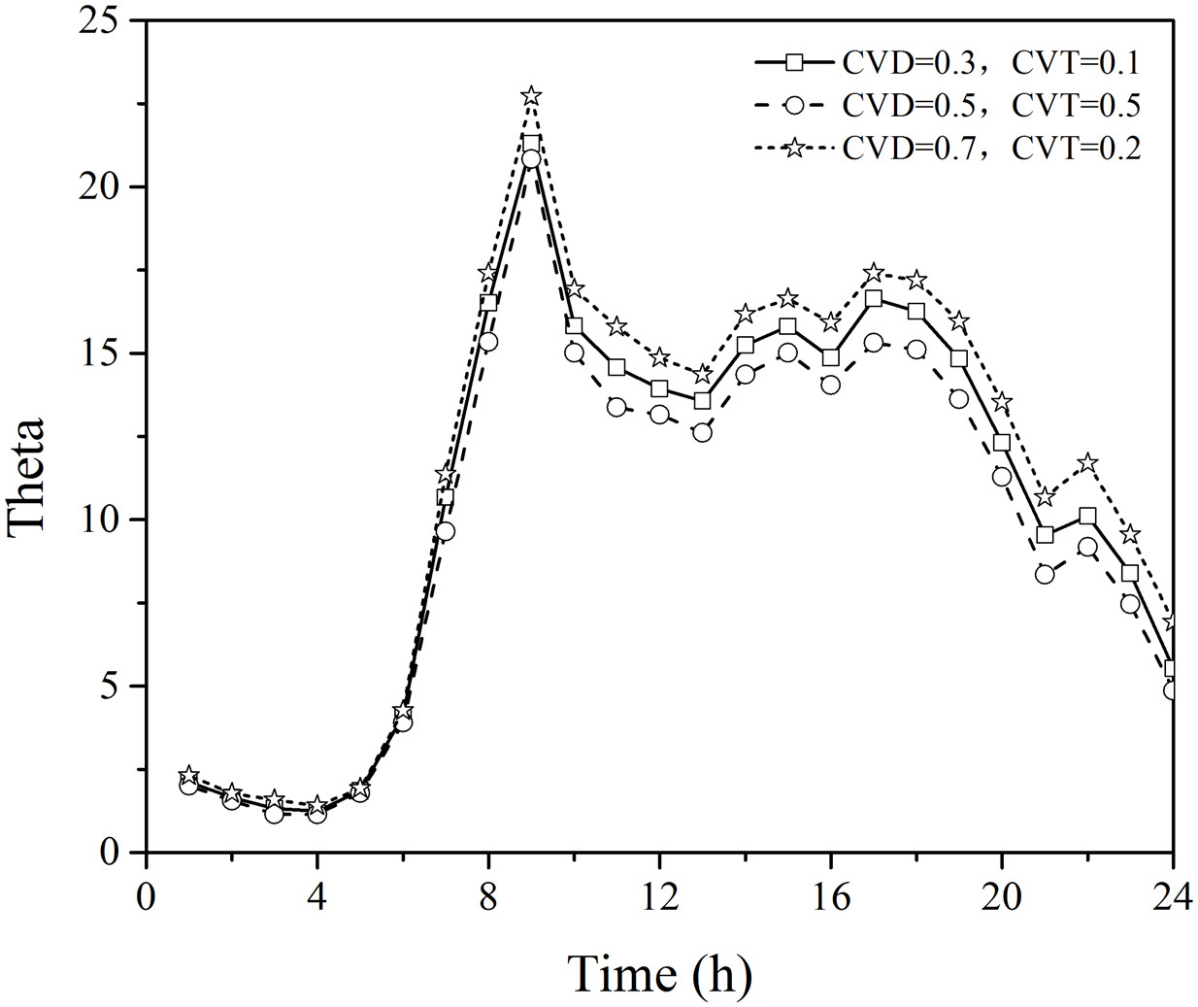
Estimated Theta values in the actual network by time of day.

It is interesting to observe that the estimated RMSE(OD), RMSE(LF), and Theta for both hypothetical and actual networks exhibit a similar trend yet have obvious differences. Two primary reasons may explain these differences: First, the network topology is quite different for the two scenarios. The hypothetical network is unidirectional, where each node can be either origin or destination. In contrast, the actual network is bidirectional, where each node is both origin and destination, and thus multiple paths may exist between each OD pair. For example, the traffic flows on both 1-Link 1 and 1-Link 6 contribute to the OD demands from node 1 to node 2. Second, compared with the hypothetical network with equal cost parameters for all links, a more realistic BPR link performance cost function is adopted for the actual network. In the real-world network, the parameters (e.g. free-flow travel time and link capacity) are calibrated for each link based on empirical data. That said, the sensitivity analysis for Theta produced similar results for both the hypothetical and actual networks, indicating that this parameter is not sensitive to the choice of variation coefficients. In addition, the theta estimates obtained using a range of different parameter settings exhibits a similar and regular trend over time of day as shown in Fig 12. These findings provide guidance for initial parameter selection, and offer useful insight for interpreting modeling results.

## Conclusions

This paper proposes a two-stage algorithm to simultaneously estimate origin-destination matrices and link choice proportions by incorporating a dynamic dispersion parameter into the route choice model. The dispersion parameter *θ* is of practical significance in describing travelers’ route choice decisions, but has typically been assumed constant in previous studies. Finding the optimal dispersion parameter is not a straightforward task. To address this issue, this paper presents a model calibration procedure to simultaneously estimate the dispersion parameter *θ*, link choice proportions, and OD matrix. In order to obtain the Generalized Least Square (GLS) estimators of the above listed parameters, a two-stage algorithm is proposed which integrates GLS estimation into the SUE traffic assignment procedure. The first and second stages of the algorithm are applied iteratively until the maximum relative difference presented in Step 11 is achieved, after which the estimated OD matrix, link choice proportion, and dispersion parameter *θ* can be obtained. The SQP approach based on the extended quasi-Newton method is used to search for the optimal solution in the first stage of the algorithm. The SUE traffic assignment procedure is applied to incorporate both OD matrix and link choice proportion estimation into the second stage of the algorithm, and MSA is used to obtain the equilibrium link flows.

A hypothetical network was constructed to test the performance of the proposed approach, followed by a comprehensive sensitivity analysis with 50 combinations of variation coefficient combinations *cv*_*d*_ (CVD) and *cv*_*θ*_ (CVT) to investigate the stability of the estimated OD matrix, link flows, and Theta. A comparison with two different methods described in Yang et al. [15] and Lo and Chan [16] suggests that the proposed approach can achieve superior performance in terms of RMSE (OD), RMSE (LF), and accuracy of the estimated Theta parameter. Moreover, a case study is presented using a real-world congested square network in Seattle, WA to demonstrate the practicality of the proposed approach, in which the true OD matrix and observed link flows are calculated via ground-truth traffic count data collected by loop detectors. The proposed method is shown to be robust under a range of initial parameter values. The RMSE (OD) can be reduced from 3426.9 to 23.6 at *cv*_*d*_ = 0.1 and *cv*_*θ*_ = 0.1 when traffic flows are observed on five out of eight links. In addition, the estimated dispersion parameter exhibits a consistent and regular trend by time of day for all combinations of initial parameters. For future research, the proposed approach should be tested on a network of greater complexity and size, and the impact of input data inaccuracy should be considered. Additionally, further work is needed to determine the number and location of observed links required for accurate OD estimation using the proposed approach.

## Supporting Information

S1 Dataset The dataset includes the Link Speed Data and Link Volume data, and the data were collected from loop detectors located along the freeway section (I-5, I-90, I-405 and SR 520) in Seattle area, and are retrieved via the Strategic Highway Research Program 2 (SHRP 2 program). The file named as “S1 Link Speed Data” records the average speed for all links every 20-second time interval, and the other file named as “S1 Link Volume data” records volume for all links every 20-second time interval.
(RAR)
