# Correction: A Two-Stage Algorithm for Origin-Destination Matrices Estimation Considering Dynamic Dispersion Parameter for Route Choice

**DOI:** 10.1371/journal.pone.0149827

**Published:** 2016-02-12

**Authors:** Yong Wang, Xiaolei Ma, Yong Liu, Ke Gong, Kristian C. Henrickson, Maozeng Xu, Yinhai Wang

The fifth author’s name is spelled incorrectly. The correct name is: Kristian C. Henrickson. The correct citation is: Wang Y, Ma X, Liu Y, Gong K, Henrickson KC, Xu M, et al. (2016) A Two-Stage Algorithm for Origin-Destination Matrices Estimation Considering Dynamic Dispersion Parameter for Route Choice. PLoS ONE 11(1): e0146850. doi:10.1371/journal.pone.0146850
